# Isotonic Protein Solution Supplementation Enhances Growth Performance, Intestinal Immunity, and Beneficial Microbiota in Suckling Piglets

**DOI:** 10.3390/vetsci12080715

**Published:** 2025-07-30

**Authors:** Changliang Gong, Zhuohang Hao, Xinyi Liao, Robert J. Collier, Yao Xiao, Yongju Zhao, Xiaochuan Chen

**Affiliations:** 1 College of Animal Science and Technology, Southwest University, Chongqing 400715, China; m15083461065@163.com (C.G.); hzh000602@163.com (Z.H.); 18580560020@163.com (X.L.); zhaoyongju@swu.edu.cn (Y.Z.); 2 Shandong Key Laboratory of Animal Disease Control and Breeding, Institute of Animal Science and Veterinary Medicine, Shandong Academy of Agricultural Sciences, Jinan 250100, China; xiaoyao@saas.ac.cn; 3 Department of Animal, Veterinary and Food Sciences, University of Idaho, Moscow, ID 83844, USA; rcollier@ag.arizonz.edu

**Keywords:** suckling piglets, growth performance, intestinal permeability, gut microbiota

## Abstract

Suckling is crucial for piglet intestinal development and gut health, as it improves resilience during the challenging weaning phase and promotes subsequent growth. IPS, comprising Na^+^/K^+^ ions, whey protein, and glucose, has been shown to have positive effects on animal growth and intestinal health. The objectives of this study were to assess the impact of IPS consumption on the growth performance, immunity, intestinal growth and development, and microbiota structure of suckling piglets. A total of 160 newborn piglets were randomly divided into control and IPS groups, with IPS supplementation starting from 2 to 8 days after birth and continuing until 3 days before weaning. The findings revealed that IPS boosted the body weight at 24 days by 3.6% (*p* < 0.05) and improved the body weight gain from 16 to 24 days by 15.7% (*p* < 0.05). Additionally, the jejunal villus height and villus height to crypt depth ratio in the IPS group were notably increased to 1.08 and 1.31 times (*p* < 0.05), respectively, compared to the control group. Furthermore, IPS elevated the plasma levels of IgA and IgM, reduced the plasma levels of blood urea nitrogen (BUN), and enhanced the content of secretory immunoglobulin A (SIgA) in the jejunal mucosa of suckling piglets. Furthermore, IPS upregulated the mRNA expression of tight junction proteins *GLP-2*, *ZO-1*, and *Claudin-1* in jejunal tissue, while downregulating the regulatory genes in the Toll-like pathway, including *MyD88* and *TLR-4* (*p* < 0.05). The analysis of gut microbiota indicated that IPS altered the relative abundance of gut microbes, with an increase in beneficial bacteria like *Alloprevotella* and *Bacteroides*. In conclusion, this study demonstrates that IPS supplementation enhances weaning weight, growth performance, immune function, and intestinal development in piglets, supporting the integration of IPS supplementation in the management of pre-weaning piglets.

## Simple Summary

Lactation is a critical window for nutritional intervention in piglets. Isotonic protein solution (IPS) is a nutritional supplement composed of sodium and potassium ions, glucose, and whey protein. Na^+^/K^+^ maintains intestinal ion homeostasis and promotes nutrient transport by small intestinal epithelial cells. As shown in this study, the growth performance of suckling piglets was improved by supplementing them with IPS solution at 2–8 days after birth and 3 days pre-weaning, mainly by improving barrier function, regulating gene expression, and promoting beneficial microbiota to promote growth and intestinal development in suckling piglets, making it a promising nutritional supplement for pre-weaning piglet management.

## 1. Introduction

The initial stages of postnatal piglet development are critical for the establishment of the gastrointestinal tract’s structure, the maturation of digestive enzymes, and the colonization of intestinal microbiota [1,2]. The early weeks of a piglet’s life represent a pivotal “window of opportunity” that facilitates the intestinal adaptation processes, which in turn confer lifelong growth advantages [3]. Higher weaning weights in piglets are correlated with enhanced average daily gains during the subsequent growing-finishing phases and earlier attainment of slaughter weights [4]. On large, modern pig farms, neonatal piglets face numerous stressors, including insufficient milk supply, pathogen exposure, and suboptimal management practices. These challenges can lead to compromised intestinal development, diminished immune responses, alterations in the intestinal microbiota, reduced growth performance, and increased mortality rates and incidence of diarrhea [5,6]. However, current nutritional interventions often show variable efficacy or fail to comprehensively address the multifactorial nature of these stressors. In practical pig farming, this often manifests as a high incidence of pre-weaning diarrhea, impaired immune function, and increased mortality, particularly in piglets facing early-life stressors [7,8,9]. Therefore, improving intestinal health and development during the suckling period is crucial for the long-term wellbeing of pigs.

Nutritional interventions early in life are imperative to modify the gut microbiota composition of piglets and bolster their immune systems [10,11]. Current interventions include oral supplementation (such as prebiotics and functional amino acids), enhanced milk provision, and the administration of peristaltic feeds before weaning. Research has shown that supplementation with an alkaline mineral complex in drinking water can enhance intestinal morphology, mitigate inflammatory responses, and fortify intestinal barrier function, thereby accelerating the growth performance of weaned piglets [12]. The Na^+^/K^+^ ratio plays an important role in maintaining intestinal ionic homeostasis and facilitating the transport of nutrients by small intestinal epithelial cells, such as glucose [13,14]. Studies have shown that early supplementation of isotonic protein solutions can improve the intestinal microbiota of piglets [15]. Despite these advances, there remains a need for integrated nutritional strategies that simultaneously target multiple aspects of gut health (microbiota, barrier, immunity) in the critical pre-weaning period. Consequently, we have designed an isotonic protein solution nutritional supplement designed to enhance the gut health and growth performance of pre-weaning piglets, mainly containing whey protein, Na^+^/K^+^, and glucose. This study aims to evaluate the effects of this synergistic formulation on gut microbiota composition, intestinal barrier integrity and morphology, immune markers, and growth performance in pre-weaning piglets, hypothesizing that it will enhance intestinal resilience and provide a foundation for improved lifelong productivity.

## 2. Materials and Methods

### 2.1. Animals

All experiments were conducted in strict accordance with the principles and guidelines of the Southwest University Institutional Animal Care and Use Committee (Approval No. GB14925-2010, 2019). The study adhered to the ARRIVE guidelines to ensure ethical and transparent reporting of animal research. The piglets used in the experiment, along with their feeding and management, were sourced from and handled at Guangyuan Futai Pig Farm, a commercial and standardized pig breeding facility located in Sichuan, China.

### 2.2. Study Design and Animal Diets

In this trial, 16 pregnant sows with average parities of 2–3 were selected. The sows did not have diarrhea or any other digestive disease and were not subjected to antibiotic treatment prior to the study. The sows were transferred to the farrowing room about a week before the expected farrowing date. At farrowing, the 16 sows produced over 160 newborn piglets (Duroc-Landrace-Yorkshire). Each group comprised 8 litters of piglets (10 piglets per litter). All individual piglets were measured for growth performance indicators, including body weight. However, statistical analyses were conducted at the litter level by calculating the average body weight per litter. Group differences were evaluated based on the mean values from 8 litters per group (*n* = 8 litters/group). For slaughter-related measurements (blood and intestinal tissue analyses), 6 litters were randomly selected from each group (1 piglet per selected litter), with piglets chosen to represent their respective litters. Specifically, only piglets whose body weights deviated by no more than 5% from the litter average were selected for sampling. The resulting data were treated as litter-level measurements, and intergroup comparisons were performed using litters as the statistical unit (*n* = 6 litters/group). No significant differences in initial body weight were observed among litters (*p* > 0.05), and the gender ratio was evenly distributed. Both sows and their litters were housed within the same farrowing crates, with the average temperature in the production area maintained at 25–27 °C. The NRC criteria for diet formulation for lactating sows were adhered to in our trial, and the nutrient content is detailed in Table 1. Sows were fed individually throughout the trial period and had free access to water and feed.

**Figure 1:**
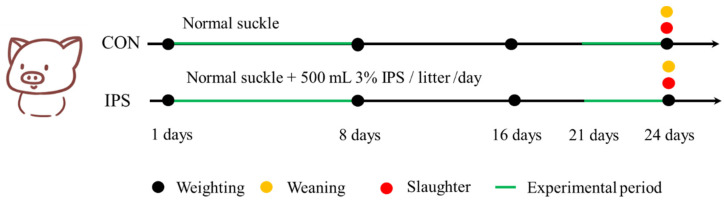
The time flow chart for treatment and data collection.

Within 24 h after farrowing, all piglets were individually marked and weighed, with live birth records documented. Litter size was standardized to 10 piglets per sow through cross-fostering, ensuring no significant inter-group difference in initial body weight and a balanced sex ratio across all litters. The piglets in the control group received no additional supplements before weaning. From days 2 to 8 of age, piglets in the IPS group were supplemented once daily with 500 mL of 3% IPS solution provided in open-top containers. Three days pre-weaning, IPS piglets received a porridge mixture consisting of creep feed and 3% IPS solution (1.5 kg solution: 1 kg dry feed). To control for feed physical form, CON piglets received an isovolumetric porridge mixture of creep feed and water (1.5 kg water: 1 kg dry feed) during the same pre-weaning period. The experimental design is summarized in Figure 1, and the nutritional composition of IPS is shown in Table 2. The 3% concentration was determined based on the osmotic pressure of mammalian plasma, which typically ranges from 290 to 310 mOsm/L. This concentration ensures an isotonic solution with an osmotic pressure of approximately 300 mOsm/L. All animals remained clinically healthy throughout the trial. Daily intake of IPS solution, porridge mixtures, and dry feed was recorded per litter.

### 2.3. Growth Performance

At 1, 8, 16 and 24 days of age, the weight of all piglets was measured, and the weighing time was consistent at each time point. The average daily gain (ADG) was calculated according to the following formula: ADG = total weight gain/(number of test days × number of tests).

### 2.4. Sample Collection

At 24 days of age, 6 piglets were randomly selected from each group. Total 12 piglets were exsanguinated after being injected with sodium pentobarbital (50 mg/kg). Whole blood samples were collected and centrifuged at 3000× *g* for 15 min in EDTA tubes and separated plasma samples were stored at −80 °C for biochemical profile analysis. The gastrointestinal tracts were dissected to extract a sample specifically from the intestine. The middle segment of the jejunum and ileum (approximately 2 cm) was fixed in 4% paraformaldehyde for intestinal morphological measurement. The intestinal mucosa from the middle segment of the jejunum was gently scraped, and both the jejunum mucosa samples and the contents of the ileocecal region were stored at −80 °C for subsequent microbiota analysis.

### 2.5. Immune and Plasma Biochemical Profiling

Plasma biochemical parameters, including urea nitrogen (BUN), glucose (GLU), total cholesterol (TC), total protein (TP), albumin (ALB), globulin (GLB), aspartate aminotransferase (AST), and alanine aminotransferase (ALT) were measured by using an automated biochemistry analyzer with the commercial kits (SMT-120VP, Seamaty Technology, Chengdu, China).

Plasma immunoglobulin A (IgA), immunoglobulin G (IgG), immunoglobulin M (IgM) and jejunal mucosal secretory immunoglobulin A (SIgA) were measured by commercial porcine ELISA kits (RX500986P, RX500984P, RX500977P, RX501029P, Ruixin Biological Technology, Quanzhou, China). In addition, the content of secreted SIgA was analyzed with the supernatant that was obtained from the jejunal mucosal homogenate after centrifugation (3000× *g*, 10 min, 4 °C).

### 2.6. Intestinal Morphology Analysis

Jejunal and ileal intestinal samples were fixed with 4% paraformaldehyde, embedded with paraffin, and stained with hematoxylin and eosin. Tissue section (6 μm thickness) images were captured by using a digital trinocular camera microscope (BA210 LED Digital, Motic Microscopes, Xiamen, China). Measurements of intraseptal intestinal villi height (VH) and crypt depth (CD) were analyzed using Motic Images Advanced 3.2 software, allowing for the calculation of the V/C. Each sample from each experimental group was examined for at least 10 well-oriented intact villi and their associated crypts.

### 2.7. RNA Extraction and RT-qPCR Analysis

Total RNA was extracted from the jejunal by the TRIZOL method. The concentration and purity of RNA were determined by Nanodrop™ One spectrophotometer (Thermo Fisher Scientific, Waltham, MA, USA). The mRNA was then reverse-transcribed to cDNA using the PrimeScript™ RT kit and gDNA Eraser (RR047A, TAKARA, Beijing, China). The extracted mRNA was spectrophotometrically quantified (OD_260_/OD_280_: 1.8–2.2). The reverse-transcribed cDNA was then stored in a refrigerator at −20 °C for later use. The relative expression of mRNA was measured using TB Green Premix Ex Taq™ II (RR820A, TAKARA) with the CFX96 Touch™ Real-time PCR Detection system (Bio-Rad, Hercules, CA, USA). The RT-PCR thermal cycling conditions were 95 °C for 30 s; 95 °C for 5 s; 60 °C for 30 s for 40 cycles. The relative level of mRNA expression was calculated by using the 2^−ΔΔCt^ method after normalization with *β-actin* as a housekeeping gene. The primers used in this study are listed in Table S1.

### 2.8. Microbiome Analysis

The microbial communities in the jejunum and cecum of piglets were analyzed using high-throughput 16S rDNA sequencing technology (Gene Denovo, Guangzhou, China). Genomic DNA was extracted from the contents of jejunum and cecum samples, followed by amplification of the V3–V4 hypervariable region of the 16S rDNA using barcode-specific primers. The primer sequences used were 341F: CCTACGGGNGGCWGCAG and 806R: GGACTACHVGGGTATCTAAT. The purified amplicons were ligated to sequencing adapters to construct a paired-end sequencing library, which was then sequenced on an Illumina NovaSeq 6000 platform (PE250, Illumina, San Diego, CA, USA). Finally, the generated 16S rDNA sequencing data were processed and analyzed using the Omicsmart online analysis tool.

### 2.9. Statistical Analysis

All data were analyzed using GraphPad Prism 10.4 software (GraphPad Software, San Diego, CA, USA). Independent sample *t*-tests were performed to assess inter-group differences in mean values. Prior to conducting t-tests, the Shapiro–Wilk procedure was employed to evaluate data normality and homogeneity of variance. When the assumption of normality was not met, non-parametric alternatives, such as the Mann–Whitney U test, were applied. Welch’s *t*-test and the Wilcoxon rank test were used to compare α diversity indices (Chao1, Shannon, Simpson, and ACE) across groups. All data are presented as mean values, the standard error of mean (SEM) represented variation in data, and a value of *p* < 0.05 was considered statistically significant.

## 3. Results

### 3.1. Growth Performance

Between 2 and 8 days of age, the daily intake of IPS per litter is usually 95%. Three days pre-weaning, the average daily intake of the IPS-supplemented porridge mixture (3% IPS solution) in the IPS group was 0.230 kg/pig (dry matter basis), while the control group received a water-based porridge mixture at 0.228 kg/pig (dry matter basis), with no significant difference between groups (*p* > 0.05).

In this trial, a total of 160 newborn piglets (initial weight 1.51 ± 0.21 kg) were used to assess the impact of IPS supplementation on piglet growth performance. Overall, piglets supplemented with IPS demonstrated a 250 g increase in body weight by day 24 and an increase in ADG in piglets receiving IPS from day 16 to day 24 compared to the CON (Table 3; *p* < 0.05). However, no differences were observed in body weight and ADG between IPS-supplemented piglets and the control group during days 1 to 8 and days 8 to 16.

### 3.2. Biochemistry Parameters

Blood biochemical parameters are reliable indicators of systemic growth and metabolic status. In this study, IPS supplementation did not exert a detectable effect on plasma-associated enzymes (albumin, globulin, aspartate aminotransferase, alanine aminotransferase), immune-related indices (total protein), and metabolites (glucose, total cholesterol, triglycerides) compared to the CON. However, it is noteworthy that the blood urea nitrogen content of piglets in the IPS supplementation group decreased by 18.79% compared to the CON (Table 4; *p* < 0.05). This reduction suggests a potential modulation of nitrogen metabolism due to IPS supplementation.

**Figure 2:**
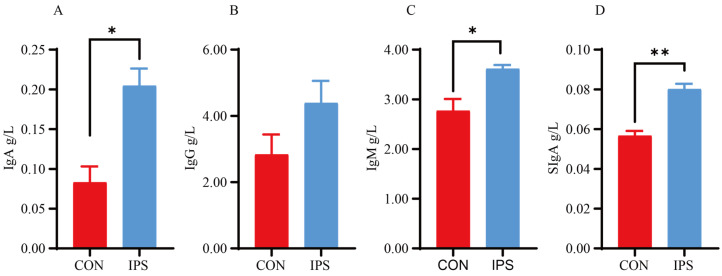
Effect of IPS on plasma and jejunal mucosal immunoglobulins in suckling piglets (*n* = 6). (**A**) Plasma immunoglobulin A (IgA); (**B**) plasma immunoglobulin G (IgG); (**C**) plasma immunoglobulin M (IgM); (**D**) secretory immunoglobulin A (SIgA) of the jejunal mucosa. Data are presented as mean ± SEMs; * *p* < 0.05, ** *p* < 0.01.

The concentration of immunoglobulins in the blood serves as a comprehensive indicator of the body’s immune status. In this study, the IgA and IgM levels in the IPS group were 1.65 and 1.27-fold higher than those in the control group, respectively (Figure 1; *p* < 0.05). However, there were no differences observed in IgG levels between the two groups. The immune protein SIgA is recognized as a key antibody in the mucosal immune barrier, playing a crucial role in specific immunity and immunomodulation. Analysis of SIgA content on the mucosal surface of the jejunum indicated that the SIgA content in the IPS group was 1.41 times higher than that in the CON (Figure 2; *p* < 0.05). This suggests that IPS supplementation may enhance mucosal immune function, potentially contributing to improved immune defense mechanisms in piglets.

### 3.3. Intestinal Morphological

**Figure 3:**
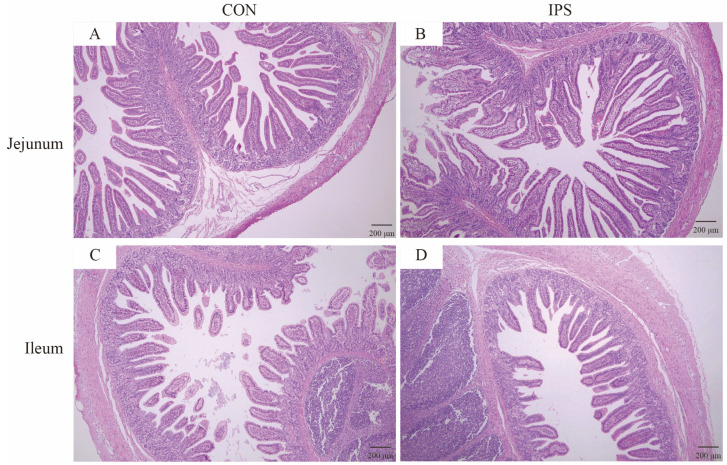
Effect of IPS on intestinal morphology of jejunum and ileum in suckling piglets (*n* = 6). (**A**) Jejunum architecture in the control group (CON); (**B**) jejunum architecture in the treatment group (IPS); (**C**) ileum architecture in the control group (CON); (**D**) ileum architecture in the treatment group (IPS); scale bar, 200 μm.

Intestinal morphology was assessed in both CON and IPS-treated piglets. The IPS group showed notable increases in jejunal villus height and villus height to crypt depth ratio, by 1.08 and 1.31 times, compared to the control group (Table 5, Figure 3; *p* < 0.05). However, in the ileum, there were no differences observed in VH, CD, and V/C between the two groups. These findings suggest that IPS supplementation may exert its growth-promoting effects, at least in part, through improving the morphological structure of the jejunum, potentially facilitating nutrient absorption and metabolism.

### 3.4. Gene Expression of Immune, Gut Growth and Tight Junction Proteins

**Figure 4:**
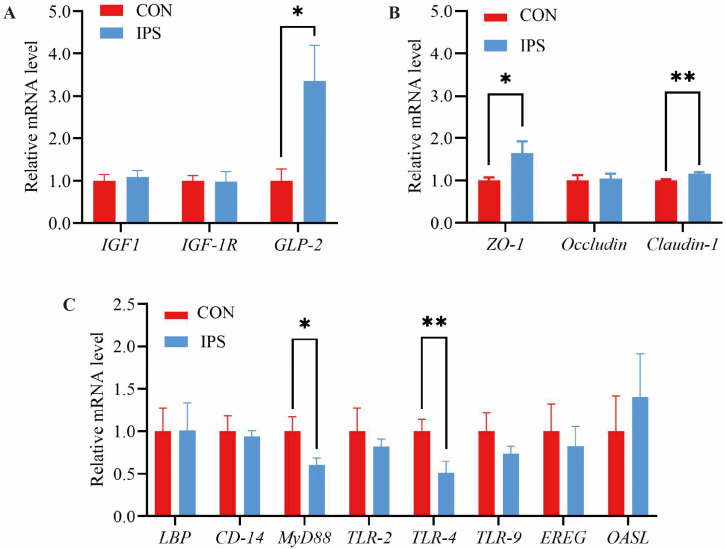
Expression of jejunum-related genes (*n* = 6). (**A**) Expression of genes related to intestinal development; (**B**) expression of intestinal barrier-related genes; (**C**) Expression of intestinal immune-related genes. Data are presented as mean ± SEMs; * *p* < 0.05, ** *p* < 0.01.

In order to understand the mechanism of IPS on intestinal development, we investigated the growth factors, immune factors, and tight junctions. The expression levels of *GLP-2* mRNA in the jejunum of the IPS group were 3.35 times higher than that of the CON (Figure 4A; *p* < 0.05). Conversely, we failed to detect differences in the expression levels of *IGF-1* and *IGF-1R* in the jejunum between the two groups. These findings suggest that IPS may influence growth performance, at least partially, by modulating the expression of genes associated with immune response and intestinal development.

Compared with CON, the expression of jejunal *ZO-1* and *Claudin-1* mRNA in the IPS group was enhanced, increasing by 1.64 and 1.56 times, respectively (Figure 4B; *p* < 0.05), compared to the values for CON. Finally, we failed to detect a difference in the mRNA expression level of *Occludin* in the jejunum between the two groups.

The mRNA expression of *MyD88* and *TLR-4* in the IPS group decreased by 36.68% and 37.12%, respectively, in contrast to CON (Figure 4C; *p* < 0.05). However, no differences were detected in the expression of *CD-14*, *TLR-2*, *TLR-9*, *EREG*, and *OASL* mRNA in the jejunum between the two groups.

### 3.5. Intestinal Microbiota Diversity and Composition

#### 3.5.1. Intestinal Microbiota Diversity

**Figure 5:**
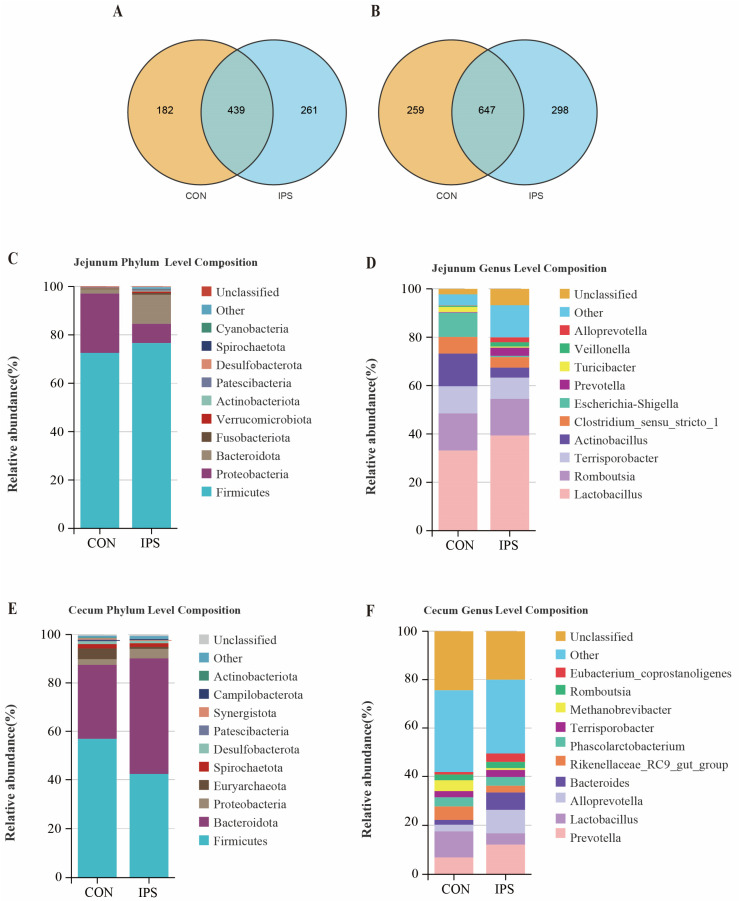
Effect of IPS supplementation on the composition of intestinal microbiota in suckling piglets (*n* = 5). (**A**) Venn diagram illustrating the differentially abundant OTUs in the jejunum; (**B**) Venn diagram illustrating the differentially abundant OTUs in the caecum; (**C**) analysis of microbial abundance at the jejunal phylum level; (**D**) analysis of microbial abundance at the jejunal genus level; (**E**) analysis of microbial abundance at the caecum phylum level; (**F**) analysis of microbial abundance at the caecum genus level.

Based on the optimized sequencing depth (99% coverage), the OTU (Operational Taxonomic Units) clustering analysis revealed the following findings (Table S1). In the jejunum, the CON group on average yielded 621 operational taxonomic units (OTUs), while the IPS group had 700 OTUs. There were 439 OTUs shared by both groups (Figure 5A). In the caecum, the CON group obtained an average of 906 OTUs, and the IPS group achieved 945 OTUs. A total of 647 OTUs were common to both groups (Figure 5B).

In the realm of intestinal microbiota ecological research, α-diversity analysis serves as a fundamental metric for evaluating the stability and functional potential of the host intestinal flora. It quantifies the number of microbial taxonomic units and the balance of their distribution within samples using the Chao1 index (reflecting species richness) and the Shannon index (indicating species evenness). In this study, no significant differences in α-diversity were detected among the groups in either the jejunum or the cecum (Table 6). This suggests that the intervention of isotonic protein solution did not significantly affect the overall species abundance or the degree of community homogenization of the cecal microbiota in suckling piglets.

In this study, we employed principal coordinate analysis (PCoA) and principal component analysis (PCA) to evaluate the regulatory effects of the isosmotic protein solution (IPS) on the intestinal microbiota structure in suckling piglets. Results demonstrated partial overlap between IPS and control groups in jejunal microbiota, whereas distinct separation was observed in cecal microbiota, indicating heightened sensitivity of cecal microbial communities to IPS intervention. This differential response may be attributed to the functional complexity of the cecum as the primary microbial colonization site (Figure S2).

#### 3.5.2. Alterations in Jejunal Microbiota Structure

Jejunal microbiota analysis revealed significant shifts in dominant phyla between CON and IPS groups. CON was dominated by *Firmicutes* and *Proteobacteria*, collectively representing 97.06% of microbiota. Under IPS intervention, *Firmicutes* increased to 76.59% while *Proteobacteria* substantially decreased to 7.96%, with *Bacteroidota* significantly rising from 1.79% to 12.04% to become the third dominant phylum. Minor phyla showed 8.4-fold and 3.6-fold increases in *Verrucomicrobiota* and *Actinobacteriota*, respectively, under IPS, while *Fusobacteriota* reached 0.72%. All other phyla remained below 0.5% in both groups (Figure 5C).

To further explore the specific changes at the genus level, an in-depth analysis was performed. The jejunal microbiota of piglets exhibited significant differences in the distribution of dominant bacterial groups between the CON group and the IPS group. In the CON group, *Lactobacillus*, *Romboutsia*, *Terrisporobacter*, and *Actinobacillus* were the predominant genera, with Firmicutes-related genera accounting for 67.6% of the total (including *Lactobacillus* and *Romboutsia*). By contrast, in the IPS group, the abundance of *Lactobacillus* increased significantly to 39.32%, while *Actinobacillus* and *Escherichia-Shigella* showed a marked decrease. Notably, *Prevotella* and *Alloprevotella*, belonging to the *Bacteroidota* phylum, increased more than tenfold in the IPS group compared to the CON group (Figure 5D).

#### 3.5.3. Alterations in Caecum Microbiota Structure

The analysis of the microbial community in the caecum of piglets revealed significant differences in the abundance of major bacterial groups between the CON group and the IPS group. In the CON group, *Firmicutes* and *Bacteroidota* were predominant. In contrast, in the IPS group, the abundance of *Bacteroidota* significantly increased to 47.76%, while *Firmicutes* decreased to 42.32%. Among secondary bacterial groups, *Proteobacteria* showed a slight increase in the IPS group, whereas the abundance of *Euryarchaeota* significantly decreased. The fluctuations in other bacterial groups, such as *Spirochaetota* and *Desulfobacterota*, remained below 1.5% between the two groups (Figure 5E).

Analysis of the cecal microbiota at the genus level revealed distinct structural differences between the CON group and the IPS group. In the CON group, *Lactobacillus*, *Prevotella*, and *Rikenellaceae_RC9_gut_group* were the most abundant genera. However, in the IPS group, there was a significant enrichment of *Bacteroidota*-related genera, with *Prevotella*, *Alloprevotella*, and *Bacteroides* collectively accounting for 28.75%. Furthermore, the abundance of the methanogenic archaeon *Methanobrevibacter* decreased by 82.3%, and *Lactobacillus* abundance dropped by 56.4%. Of particular interest, the butyrate-producing genus *Eubacterium* coprostanoligenes increased by 2.5 times in the IPS group (Figure 5F).

### 3.6. Spearman Correlation Analysis of Intestinal Microbiota

**Figure 6:**
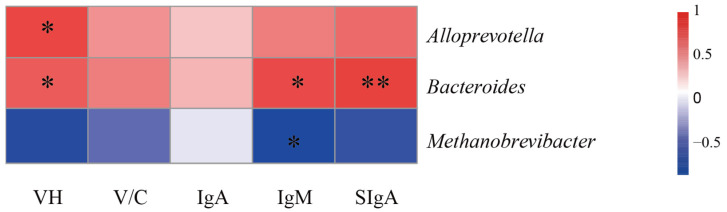
Spearman correlation analysis of intestinal microbiota; * *p* < 0.05, ** *p* < 0.01.

Further exploration revealed significant associations between differential gut microbiota and both piglet immunity and gut growth. *Alloprevotella* exhibited a positive correlation with jejunal VH (Figure 6; *p* < 0.05). *Bacteroides* demonstrated positive correlations with jejunal VH, serum immunoglobulin IgM levels, and jejunal mucosal SIgA (*p* < 0.05). Conversely, *Methanobrevibacter* displayed a negative correlation with serum immunoglobulin IgM levels (*p* < 0.05).

## 4. Discussion

This study investigated the effects of IPS as a supplement on various aspects of suckling piglets’ health and development. This research identifies the beneficial effects of IPS on growth performance, immune functionality, intestinal development and health, integrity of the intestinal barrier, and the structure of the microbiota. Nutritional supplements, particularly prebiotics and lactoferrin, have attracted considerable scientific interest for their potential to enhance both growth performance and gastrointestinal health in suckling piglets [16]. In this investigation, IPS composed of whey protein, glucose, and Na^+^/K^+^ ions, was shown to improve the growth metrics of suckling piglets, as evidenced by increases in weaning weight and average daily gain. Crucially, the observed enhancements in growth performance were concomitant with advancements in intestinal development and health, thereby elucidating a potential mechanism through which IPS may exert its beneficial effects on piglet health and growth.

Weaning weight serves as a critical metric for assessing the robustness of suckling piglets and is directly correlated with the economic productivity of swine operations [17]. Our findings indicate that IPS elevated both the weaning weight and the daily gain of the piglets. Previous research has demonstrated that the inclusion of whey protein in diets can enhance the growth metrics of preterm piglets and foster improvements in intestinal development and immune function [18]. Additionally, previous research has reported that glyco-electrolyte supplements administered to weaned piglets not only improved growth performance but also improved intestinal integrity. Higher weaning weights have a significant impact on pig lifetime growth and slaughter performance [19]. Although the control lacked an iso-energetic substitute, the negligible caloric contribution of IPS (1.5 g/day/piglet) indicates observed effects stem primarily from bioactive nutrients. In summary, our study found that IPS supplementation positively affected growth performance and weaning weight in suckling piglets.

Blood biochemical indices reflect an animal’s growth performance and metabolic activity [20]. Results of this study demonstrated a significant decrease of 18.79% in blood urea nitrogen concentrations following IPS supplementation. BUN levels are recognized as a biomarker for protein utilization in animals, with an inverse relationship observed between BUN concentrations and both protein utilization efficiency and average daily gain [21,22,23]. These findings are consistent with the outcomes of our experiments, suggesting that IPS supplementation potentially enhances protein absorption and utilization efficiency in piglets, thereby improving their overall growth performance.

Gut morphology serves as a pivotal indicator of intestinal health [24], with the first week post-partum being a critical developmental window for piglet intestines [25]. Small intestinal villi, essential for nutrient digestion and absorption [23], undergo rapid maturation immediately after birth [26]. Within the small intestine, parameters such as VH, CD, and the V/C are indicative of the health and functional absorption efficiency of the intestine. An increase in the V/C is associated with enhanced nutrient and fluid absorption [27,28], while increased VH directly improves digestive capacity and growth performance [26]. In this study, IPS supplementation significantly increased jejunal VH and V/C ratio in piglets. Furthermore, other studies have documented the beneficial effects of whey protein and ionized water supplementation on the morphology of the small intestine in piglets [29,30]. Notably, the increased VH, V/C ratio directly correlates with the 15.7% ADG gain (Days 16–24), indicating enhanced nutrient absorption efficiency.

The structural development of the gastrointestinal tract is governed by several key developmental genes, notably *IGF-1*, *IGF-1R*, and *GLP-2*, which are significant markers of cell differentiation and intestinal development [31]. Our investigation observed a marked upregulation of *GLP-2* expression in the jejunal region following IPS supplementation. As an intestinal peptide hormone, elevated *GLP-2* levels can induce a range of beneficial effects on gut health and functionality [32]. *GLP-2* is known to promote mucosal growth and cellular proliferation within the intestine [33]. This proliferation and the associated increase in intestinal mucosal cell count contribute to the expansion of the intestinal surface area, elevation of VH, and facilitation of nutrient absorption [34]. Concurrently, our experiments observed an increase in VH within the jejunum of piglets. These observations suggest that IPS supplementation may induce modifications in intestinal mucosal morphology, thereby promoting intestinal development, enhancing nutrient absorption, and ultimately augmenting piglet growth performance.

The intestinal barrier serves as a critical protector of intestinal health, safeguarding against deleterious agents such as bacteria, endotoxins, and antigens [35]. Particularly during the early stages of piglet development, the intestinal barrier undergoes maturation, characterized by a relatively thin mucus layer and a concurrent gradual maturation of the immune system, coinciding with the rapid growth of the intestinal tract [36,37]. Tight junctions are integral components of the intestinal barrier, forming its physical barricade [35,38]. Crucial elements of the intestinal mucosal epithelial barrier, including *ZO-1*, *Claudin-1*, and *Occludin*, are intimately associated with intestinal permeability [39,40]. This study demonstrated an increase in the mRNA expression of *ZO-1* and *Claudin-1* genes in the jejunum of piglets subsequent to IPS supplementation. This finding is consistent with the research by Chen et al., who reported that the addition of an AMC to drinking water could enhance the expression levels of small intestinal tight junction factors in weaned piglets, thereby improving intestinal barrier function and overall intestinal health. Thus, our results indicate that IPS supplementation may strengthen jejunal barrier function in piglets, potentially contributing to enhanced intestinal health and functionality.

The susceptibility of the gastrointestinal tract in suckling piglets to pathogenic bacterial infections underscores the importance of enhancing their immune defenses to mitigate the incidence of diarrhea and improve survival rates [41]. Immunoglobulins serve as critical markers of immune competence, playing an essential role in bolstering immunity [42,43]. Guo et al. have highlighted the significance of IgA, IgG, and IgM in serum as primary components of humoral immunity, bolstering monocyte macrophage phagocytosis and impeding the proliferation of pathogenic microorganisms [44,45,46]. IgA, with its predominant presence in mucous membranes and serum, plays a crucial role in defending against pathogenic invasions [47]. Within the intestinal mucosa, IgA exists as a dimer, termed SIgA, which serves as the primary defender in intestinal antigen-specific immunity [48,49]. In our study, the supplementation of IPS notably elevated the serum levels of IgA, IgM, and SIgA in suckling piglets.

Toll-like receptors are pivotal components of innate immunity, essential for recognizing pathogens and initiating immune responses. These receptors act as a bridge between innate and adaptive immunity and play a regulatory role in various immune cells and factors [50]. This study evaluated the impact of IPS supplementation on the expression of TLR pathway-related genes within the jejunal mucosa of piglets. The results revealed that IPS supplementation induced a downregulation of gene expression associated with the *TLR-4* and *MyD88* pathways in the jejunum of suckling piglets. The downregulation of TLR-4/MyD88 signaling may reduce pro-inflammatory cytokine production. Coupled with elevated plasma IgA/IgM, this suggests that IPS optimizes immune balance by mitigating excessive inflammation.

The gut microbiome plays an integral role in maintaining intestinal health, facilitating essential functions such as nutrient absorption, metabolic processes, resistance against pathogenic invasion, and the reinforcement of the intestinal immune barrier [51,52,53]. To validate the functional association between physiological improvements and IPS-induced microbiota restructuring, we performed 16S rDNA sequencing analysis on the contents of both the jejunum and cecum. This study documented a significant alteration at the phylum level in the gut microbiome following IPS supplementation, characterized by decreased relative abundance of *Firmicutes* and *Euryarchaeota*, alongside an increased prevalence of *Bacteroidota*. *Firmicutes* and *Bacteroidota* represent two dominant phyla within the intestinal microbiota of animals [54], *Bacteroides* as a core genus within the phylum *Bacteroidota*, serving as a key player in the breakdown of dietary carbohydrates and polysaccharides. This metabolic function not only enhances nutrient utilization but also promotes intestinal mucosal development, facilitates immune system maturation, strengthens host immune defenses, and contributes to the maintenance of intestinal microecological homeostasis [55,56]. Furthermore, these findings indicate a positive correlation between the presence of *Bacteroides* and various indicators of intestinal health, including jejunal VH, plasma IgM, and SIgA concentrations in the jejunal mucosa. Recent studies emphasize the essential role of intestinal *Bacteroides*, a gram-negative bacterial genus that dominates the gut microbiota, in sustaining mucosal immune function. These bacteria produce butyrate, which not only activates T cell-mediated immune responses and restricts pathogen colonization but also promotes the expression of plasma cell-derived SIgA in the intestinal mucosa [57,58].

At the genus level, IPS supplementation significantly decreased *Methanobrevibacter*, a methanogenic archaeon involved in gut microbial balance but paradoxically associated with inflammatory initiation [59,60], while increasing the abundance of *Alloprevotella* and *Bacteroides*. These microbial shifts are functionally linked to enhanced butyrate and propionate production, which activate *FFAR2* receptors and subsequently upregulate tight junction proteins such as *ZO-1* and *Claudin-1* [61,62]. These changes correlate directly with physiological improvements; *Alloprevotella* shows a positive association with jejunal VH, while *Bacteroides* correlates with VH, plasma IgM, and mucosal SIgA levels [63,64,65]. In contrast, *Rikenellaceae_RC9_gut_group*, which is enriched in pigs with low feed efficiency and high-fat diet models, appears to exert detrimental effects on growth performance [66]. Meanwhile, the reduced abundance of *Christensenellaceae_R-7_group* aligns with lower fiber intake and iron absorption requirements during lactation [67,68], a nutrient essential for intestinal villi development [69]. Collectively, *Alloprevotella* (butyrate producer) and *Bacteroides* (propionate producer) act synergistically to reinforce intestinal barrier integrity and immune function, suggesting that IPS enhances piglet growth by modulating gut microbiota composition.

## 5. Conclusions

This study shows that IPS improves growth performance and intestinal health in suckling piglets. IPS strengthens intestinal barrier integrity through upregulation of tight junction proteins and *GLP-2*-mediated mucosal development, while modulating gut immunity via elevated SIgA/IgM levels and suppression of TLR-4/MyD88 signaling. Furthermore, IPS enriches beneficial microbiota, with increased *Alloprevotella* abundance correlating with VH and *Bacteroides* enrichment linked to SIgA production. However, this study is limited to the pre-weaning phase, and long-term effects post-weaning as well as detailed mechanisms of microbial metabolite interactions (e.g., SCFAs) require further investigation. Collectively, these synergistic effects establish IPS as a novel nutritional strategy for improving intestinal health in suckling piglets.

## Figures and Tables

**Table 1:** Composition of the basal diet for sows (as-fed basis).

Ingredients (%)	Content	Nutrient Level/kg ^2^	
Corn	54.50	Gross energy, MJ/kg	16.84
Soybean oil	1.50	Crude protein, %	15.63
Soybean meal	24.00	Crude fat, %	4.72
Imported fish meal	2.50	Crude fibre, %	2.12
Whey	7.50	Crude ash, %	3.82
Paddy	6.00	Lys, %	1.17
Premix ^1^	4.00	Thr, %	0.74
Total	100	Ile, %	0.53
		Ca, %	0.87
		TP, %	0.57

^1^ Supplied per kilogram of diet: Vitamin A, 10,000 IU; Vitamin E, 20.00 mg; Vitamin D, 3800 IU; Vitamin K 5.00 mg; folic acid, 2.50 mg; pantothenic acid, 12.00 mg; biotin, 0.12 mg; Ca, 2.32 g; Na, 6.75 g; Cu, 20.48 g; Zn, 87.15 mg; Mn, 32.54 mg. ^2^ The nutrient levels were calculated values.

**Table 2:** The calculated values of nutrient levels of IPS.

Ingredients	Content (%)	Nutritional Components	Content
Glucose	61.0	Crude protein	16.98
Whey protein	21.0	Crude fat	0.7
Citric acid	5.2	crude fiber	0.5
Na	5.2	crude ash	17.5
K	5.3	Lys	0.45
Sodium glutamate	1.8	Arg	0.05
Sodium benzoate	0.5	Lle	0.07
Total	100		

**Table 3:** Effects of IPS on growth performance of suckling piglets (*n* = 8).

Item	CON	IPS	*p*-Value
BW, kg			
Day 1	1.55 ± 0.22	1.46 ± 0.21	0.440
Day 8	3.01 ± 0.26	2.91 ± 0.43	0.575
Day 16	5.05 ± 0.25	4.99 ± 0.33	0.699
Day 24	6.95 ± 0.29	7.20 ± 0.11	0.045
ADG, g/d			
Day 1–8	182.60 ± 41.26	181.25 ± 33.83	0.968
Day 8–16	254.02 ± 37.29	259.71 ± 28.52	0.921
Day 16–24	238.52 ± 18.48	276.11 ± 29.71	0.038
Day 1–24	225.05 ± 19.23	239.02 ± 6.67	0.102

**Table 4:** Effect of IPS on serum biochemistry of suckling piglets (*n* = 6).

Item	CON		IPS		*p*-Value
	Mean	CV%	Mean	CV%	
TP, g/L	60.74 ± 5.90	8.60	58.19 ± 3.77	6.40	0.431
ALB, g/L	37.48 ± 3.47	8.30	35.98 ± 2.53	7.00	0.410
GLB, g/L	23.28 ± 2.96	11.70	22.23 ± 1.55	6.90	0.601
AST, U/L	69.83 ± 9.11	12.90	61.20 ± 5.85	9.50	0.431
ALT, U/L	49.83 ± 14.18	22.30	48.29 ± 13.45	19.50	0.990
BUN, mmol/L	4.79 ± 0.48	10.30	3.89 ± 0.85	5.70	0.017
GLU, mmol/L	6.63 ± 0.71	13.20	6.89 ± 0.81	11.70	0.413
TC, mmol/L	3.08 ± 0.74	19.00	3.19 ± 0.64	20.00	0.609

**Table 5:** Effect of IPS on the intestinal morphology of suckling piglets (*n* = 6).

Item	CON	IPS	*p*-Value
Jejunum			
VH, μm	601.61 ± 15.96	650.71 ± 25.44	0.026
CD, μm	130.90 ± 27.24	106.70 ±14.12	0.188
VH:CD	4.73 ± 0.78	6.18 ± 0.80	0.037
Ileum			
VH, μm	464.02 ± 44.28	491.99 ± 53.86	0.713
CD, μm	115.23 ± 10.03	128.10 ± 21.10	0.363
VH:CD	4.05 ± 0.45	3.93 ± 0.74	0.826

**Table 6:** Effect of IPS on the diversity and richness of bacterial communities in the cecum of lactating piglets (*n* = 5).

Item	CON	IPS	*p*-Value
Jejunum			
Shannon	3.57 ± 0.59	4.45 ± 1.43	0.182
Chao	832.28 ± 88.59	794.90 ± 53.70	0.784
Simpson	0.82 ± 0.08	0.87 ± 0.07	0.821
ACE	886.31 ± 96.43	834.20 ± 52.58	0.623
Cecum			
Shannon	6.37 ± 0.47	6.56 ± 0.45	0.534
Chao	1167.64 ± 145.43	1267.34 ± 100.15	0.246
Simpson	0.97 ± 0.01	0.97 ± 0.01	0.798
ACE	1250.01 ± 149.14	1357.52 ± 107.86	0.231

## Data Availability

The data that support the findings of this study are available on request from the corresponding author.

## Supplementary Materials

The following supporting information can be downloaded at: https://www.mdpi.com/article/10.3390/vetsci12080715/s1. Table S1: Sequences for primers used in quantitative real-time PCR. Table S2: Quality control statistics of 16S rDNA sequencing data from jejunum and caecum in piglets. Figure S1: Microbial diversity analysis.

## Abbreviations

The following abbreviations are used in this manuscript:

ADG	Average daily gain
ALB	Albumin
ALT	Alanine transaminase
AST	Aspartate aminotransferase
BUN	Blood urea nitrogen
BW	Body weight
CD	Crypt depth
TC	Total cholesterol
TP	Total protein
VH	Villous height

## References

[1] Ferrari C.V., Sbardella P.E., Bernardi M.L., Coutinho M.L., Vaz I.S., Wentz I., Bortolozzo F.P. (2014). Effect of birth weight and colostrum intake on mortality and performance of piglets after cross-fostering in sows of different parities. Prev. Vet. Med..

[2] Saladrigas-García M., Durán M., D’Angelo M., Coma J., Pérez J.F., Martín-Orúe S.M. (2022). An insight into the commercial piglet’s microbial gut colonization: From birth towards weaning. Anim. Microbiome.

[3] Jayaraman B., Nyachoti C.M. (2017). Husbandry practices and gut health outcomes in weaned piglets: A review. Anim. Nutr..

[4] He Y., Deen J., Shurson G.C., Wang L., Chen C., Keisler D.H., Li Y.Z. (2016). Identifying factors contributing to slow growth in pigs. J. Anim. Sci..

[5] McCance R.A. (1974). The effect of age on the weights and lengths of pigs’ intestines. J. Anat..

[6] Choudhury R., Middelkoop A., de Souza J.G., van Veen L.A., Gerrits W.J.J., Kemp B., Bolhuis J.E., Kleerebezem M. (2021). Impact of early-life feeding on local intestinal microbiota and digestive system development in piglets. Sci. Rep..

[7] Koketsu Y., Iida R., Piñeiro C. (2021). Five risk factors and their interactions of probability for a sow in breeding herds having a piglet death during days 0–1, 2–8 and 9–28 days of lactation. Porc. Health Manag..

[8] Zhang Y., Chen Y., Zhou J., Wang X., Ma L., Li J., Yang L., Yuan H., Pang D., Ouyang H. (2022). Porcine Epidemic Diarrhea Virus: An Updated Overview of Virus Epidemiology, Virulence Variation Patterns and Virus-Host Interactions. Viruses.

[9] Farmer C., Edwards S.A. (2022). Review: Improving the performance of neonatal piglets. Animal.

[10] Quigley E.M.M. (2019). Prebiotics and Probiotics in Digestive Health. Clin. Gastroenterol. Hepatol..

[11] Beaumont M., Lencina C., Painteaux L., Viémon-Desplanque J., Phornlaphat O., Lambert W., Chalvon-Demersay T. (2022). A mix of functional amino acids and grape polyphenols promotes the growth of piglets, modulates the gut microbiota in vivo and regulates epithelial homeostasis in intestinal organoids. Amino Acids.

[12] Chen C., Yin Y., Tu Q., Yang H. (2018). Glucose and amino acid in enterocyte: Absorption, metabolism and maturation. Front. Biosci.

[13] Chen J., Xu Y.R., Kang J.X., Zhao B.C., Dai X.Y., Qiu B.H., Li J.L. (2022). Effects of alkaline mineral complex water supplementation on growth performance, inflammatory response, and intestinal barrier function in weaned piglets. J. Anim. Sci..

[14] Alexander A.N., Carey H.V. (1999). Oral IGF-I enhances nutrient and electrolyte absorption in neonatal piglet intestine. Am. J. Physiol..

[15] Buzoianu S.G., Firth A.M., Putrino C., Vannucci F. (2020). Early-Life Intake of an Isotonic Protein Drink Improves the Gut Microbial Profile of Piglets. Animals.

[16] Allen B., Saunders J. (2023). Malnutrition and undernutrition: Causes, consequences, assessment and management. Medicine.

[17] Cabrera R.A., Boyd R.D., Jungst S.B., Wilson E.R., Johnston M.E., Vignes J.L., Odle J. (2010). Impact of lactation length and piglet weaning weight on long-term growth and viability of progeny. J. Anim. Sci..

[18] Nielsen C.H., Hui Y., Nguyen D.N., Ahnfeldt A.M., Burrin D.G., Hartmann B., Heckmann A.B., Sangild P.T., Thymann T., Bering S.B. (2020). Alpha-Lactalbumin Enriched Whey Protein Concentrate to Improve Gut, Immunity and Brain Development in Preterm Pigs. Nutrients.

[19] Collins C.L., Pluske J.R., Morrison R.S., McDonald T.N., Smits R.J., Henman D.J., Stensland I., Dunshea F.R. (2017). Post-weaning and whole-of-life performance of pigs is determined by live weight at weaning and the complexity of the diet fed after weaning. Anim. Nutr..

[20] Wang J., Liu S., Ma J., Dong X., Long S., Piao X. (2023). Growth performance, serum parameters, inflammatory responses, intestinal morphology and microbiota of weaned piglets fed 18% crude protein diets with different ratios of standardized ileal digestible isoleucine to lysine. Anim. Nutr..

[21] Heo J.M., Kim J.C., Hansen C.F., Mullan B.P., Hampson D.J., Pluske J.R. (2008). Effects of feeding low protein diets to piglets on plasma urea nitrogen, faecal ammonia nitrogen, the incidence of diarrhoea and performance after weaning. Arch. Anim. Nutr..

[22] Fang L.H., Jin Y.H., Do S.H., Hong J.S., Kim B.O., Han T.H., Kim Y.Y. (2019). Effects of dietary energy and crude protein levels on growth performance, blood profiles, and carcass traits in growing-finishing pigs. J. Anim. Sci. Technol..

[23] Go Y.B., Lee J.H., Lee B.K., Oh H.J., Kim Y.J., An J.W., Chang S.Y., Song D.C., Cho H.A., Park H.R. (2022). Effect of insect protein and protease on growth performance, blood profiles, fecal microflora and gas emission in growing pig. J. Anim. Sci. Technol..

[24] Gurung M., Rosa F., Yelvington B., Terry N., Read Q.D., Piccolo B.D., Moody B., Tripp P., Pittman H.E., Fay B.L. (2023). Evaluation of a Plant-Based Infant Formula Containing Almonds and Buckwheat on Gut Microbiota Composition, Intestine Morphology, Metabolic and Immune Markers in a Neonatal Piglet Model. Nutrients.

[25] Modina S.C., Aidos L., Rossi R., Pocar P., Corino C., Di Giancamillo A. (2021). Stages of Gut Development as a Useful Tool to Prevent Gut Alterations in Piglets. Animals.

[26] Wang M., Yang C., Wang Q., Li J., Huang P., Li Y., Ding X., Yang H., Yin Y. (2020). The relationship between villous height and growth performance, small intestinal mucosal enzymes activities and nutrient transporters expression in weaned piglets. J. Anim. Physiol. Anim. Nutr..

[27] Xiong X., Rao Y., Tu X., Wang Z., Gong J., Yang Y., Wu H., Liu X. (2022). Gut archaea associated with bacteria colonization and succession during piglet weaning transitions. BMC Vet. Res..

[28] Wang J., Wang W., Wang H., Tuo B. (2020). Physiological and Pathological Functions of SLC26A6. Front. Med..

[29] Monaco M.H., Wang M., Pan X., Li Q., Richards J.D., Chichlowski M., Berg B.M., Dilger R.N., Donovan S.M. (2018). Evaluation of Sialyllactose Supplementation of a Prebiotic-Containing Formula on Growth, Intestinal Development, and Bacterial Colonization in the Neonatal Piglet. Curr. Dev. Nutr..

[30] Hu P., Zhao F., Zhu W., Wang J. (2019). Effects of early-life lactoferrin intervention on growth performance, small intestinal function and gut microbiota in suckling piglets. Food Funct..

[31] Liu P., Che L., Yang Z., Feng B., Che L., Xu S., Lin Y., Fang Z., Li J., Wu D. (2016). A Maternal High-Energy Diet Promotes Intestinal Development and Intrauterine Growth of Offspring. Nutrients.

[32] Kounatidis D., Vallianou N.G., Tsilingiris D., Christodoulatos G.S., Geladari E., Stratigou T., Karampela I., Dalamaga M. (2022). Therapeutic Potential of GLP-2 Analogs in Gastrointestinal Disorders: Current Knowledge, Nutritional Aspects, and Future Perspectives. Curr. Nutr. Rep..

[33] Drucker D.J., Yusta B. (2014). Physiology and pharmacology of the enteroendocrine hormone glucagon-like peptide-2. Annu. Rev. Physiol..

[34] Wen X., Zhong R., Dang G., Xia B., Wu W., Tang S., Tang L., Liu L., Liu Z., Chen L. (2022). Pectin supplementation ameliorates intestinal epithelial barrier function damage by modulating intestinal microbiota in lipopolysaccharide-challenged piglets. J. Nutr. Biochem..

[35] Suzuki T. (2020). Regulation of the intestinal barrier by nutrients: The role of tight junctions. Anim. Sci. J..

[36] Yang S., Yang N., Huang X., Li Y., Liu G., Jansen C.A., Savelkoul H.F.J., Liu G. (2022). Pigs’ intestinal barrier function is more refined with aging. Dev. Comp. Immunol..

[37] Sun J., Chong J., Zhang J., Ge L. (2023). Preterm pigs for preterm birth research: Reasonably feasible. Front. Physiol..

[38] Minton K. (2022). Intestinal barrier protection. Nat. Rev. Immunol..

[39] Kuo W.T., Odenwald M.A., Turner J.R., Zuo L. (2022). Tight junction proteins occludin and ZO-1 as regulators of epithelial proliferation and survival. Ann. N. Y. Acad. Sci..

[40] Zhang J., Shu Z., Lv S., Zhou Q., Huang Y., Peng Y., Zheng J., Zhou Y., Hu C., Lan S. (2023). Fermented Chinese Herbs Improve the Growth and Immunity of Growing Pigs through Regulating Colon Microbiota and Metabolites. Animals.

[41] Tian M., Chen J., Wu Z., Song H., Yang F., Cui C., Chen F., Zhang S., Guan W. (2020). Fat Encapsulation Reduces Diarrhea in Piglets Partially by Repairing the Intestinal Barrier and Improving Fatty Acid Transport. Animals.

[42] El-Bohy M., Poowuttikul P., Secord E. (2019). Humoral Immune Deficiencies of Childhood. Pediatr. Clin. N. Am..

[43] Pierzynowska K., Woliński J., Weström B., Pierzynowski S.G. (2020). Maternal Immunoglobulins in Infants-Are They More Than Just a Form of Passive Immunity?. Front. Immunol..

[44] Guo H., Yao Z., Chen L., Li L., Li Y., Wang Y., Li T., Wang H., Sun L., Hao D. (2020). Humoral immune responses in piglets experimentally infected with a field strain of porcine epidemic diarrhea virus. Vet. Microbiol..

[45] Planchais C., Mouquet H. (2020). Easy pan-detection of human IgA immunoglobulins. J. Immunol. Methods.

[46] Yin Y., Wang F., Yang M., Tan B., Yin Y., Chen J., Yang Z. (2021). Lycium barbarum Polysaccharides as Antibiotic Substitutes Improve Growth Performance, Serum Immunity, Antioxidant Status, and Intestinal Health for Weaned Piglets. Front. Microbiol..

[47] de Sousa-Pereira P., Woof J.M. (2019). IgA: Structure, Function, and Developability. Antibodies.

[48] Li H., Wang D., Wu H., Shen H., Lv D., Zhang Y., Lu H., Yang J., Tang Y., Li M. (2020). SLC46A1 contributes to hepatic iron metabolism by importing heme in hepatocytes. Metabolism.

[49] Liu J., Gao R., Shi H., Cong G., Chen J., Zhang X., Shi D., Cao L., Wang X., Zhang J. (2020). Development of a rapid immunochromatographic strip test for the detection of porcine epidemic diarrhea virus specific SIgA in colostrum. J. Virol. Methods.

[50] Tartey S., Takeuchi O. (2017). Pathogen recognition and Toll-like receptor targeted therapeutics in innate immune cells. Int. Rev. Immunol..

[51] Kuziel G.A., Rakoff-Nahoum S. (2022). The gut microbiome. Curr. Biol..

[52] Singh T.P., Kadyan S., Devi H., Park G., Nagpal R. (2023). Gut microbiome as a therapeutic target for liver diseases. Life Sci..

[53] Zhou B., Yuan Y., Zhang S., Guo C., Li X., Li G., Xiong W., Zeng Z. (2020). Intestinal Flora and Disease Mutually Shape the Regional Immune System in the Intestinal Tract. Front. Immunol..

[54] Liu X., Qiu X., Yang Y., Wang J., Wang Q., Liu J., Yang F., Liu Z., Qi R. (2023). Alteration of gut microbiome and metabolome by Clostridium butyricum can repair the intestinal dysbiosis caused by antibiotics in mice. iScience.

[55] Hooper L.V., Midtvedt T., Gordon J.I. (2002). How host-microbial interactions shape the nutrient environment of the mammalian intestine. Annu. Rev. Nutr..

[56] Nolte S., Krüger K., Lenz C., Zentgraf K. (2023). Optimizing the Gut Microbiota for Individualized Performance Development in Elite Athletes. Biology.

[57] Li Y., Cao H., Zhang S., Guo P., Zhao J., Zhang D., Zhang S. (2023). Effects of the Supplementation of Essential Oil Mixtures on Growth Performance, Nutrient Digestibility, Immune Status and Microbial Community in Weaned Piglets. Animals.

[58] Jandhyala S.M., Talukdar R., Subramanyam C., Vuyyuru H., Sasikala M., Nageshwar Reddy D. (2015). Role of the normal gut microbiota. World J. Gastroenterol..

[59] Adrian M.A., Ayati B.P., Mangalam A.K. (2023). A mathematical model of Bacteroides thetaiotaomicron, Methanobrevibacter smithii, and Eubacterium rectale interactions in the human gut. Sci. Rep..

[60] Krawczyk A., Gosiewski T., Zapała B., Kowalska-Duplaga K., Salamon D. (2023). Alterations in intestinal Archaea composition in pediatric patients with Crohn’s disease based on next-generation sequencing—A pilot study. Gut Microbes.

[61] Corrêa-Oliveira R., Fachi J.L., Vieira A., Sato F.T., Vinolo M.A. (2016). Regulation of immune cell function by short-chain fatty acids. Clin. Transl. Immunol..

[62] Tan J., McKenzie C., Potamitis M., Thorburn A.N., Mackay C.R., Macia L. (2014). The role of short-chain fatty acids in health and disease. Adv. Immunol..

[63] Kong C., Gao R., Yan X., Huang L., Qin H. (2019). Probiotics improve gut microbiota dysbiosis in obese mice fed a high-fat or high-sucrose diet. Nutrition.

[64] Peng J., Tang Y., Huang Y. (2021). Gut health: The results of microbial and mucosal immune interactions in pigs. Anim. Nutr..

[65] Lan Q., Lian Y., Peng P., Yang L., Zhao H., Huang P., Ma H., Wei H., Yin Y., Liu M. (2023). Association of gut microbiota and SCFAs with finishing weight of Diannan small ear pigs. Front. Microbiol..

[66] Quan J., Cai G., Ye J., Yang M., Ding R., Wang X., Zheng E., Fu D., Li S., Zhou S. (2018). A global comparison of the microbiome compositions of three gut locations in commercial pigs with extreme feed conversion ratios. Sci. Rep..

[67] Pang K., Chai S., Yang Y., Wang X., Liu S., Wang S. (2022). Dietary forage to concentrate ratios impact on yak ruminal microbiota and metabolites. Front. Microbiol..

[68] Dong Z., Liu S., Deng Q., Li G., Tang Y., Wu X., Wan D., Yin Y. (2023). Role of iron in host-microbiota interaction and its effects on intestinal mucosal growth and immune plasticity in a piglet model. Sci. China Life Sci..

[69] Zhou J., Qin Y., Xiong X., Wang Z., Wang M., Wang Y., Wang Q.Y., Yang H.S., Yin Y. (2021). Effects of iron, vitamin A, and the interaction between the two nutrients on intestinal development and cell differentiation in piglets. J. Anim. Sci..

