# Supplementary material for: Isotonic Protein Solution Supplementation Enhances Growth Performance, Intestinal Immunity, and Beneficial Microbiota in Suckling Piglets
Source: Vet Sci. 2025 Jul 30;12(8):715. doi: 10.3390/vetsci12080715 (PMC12390416; doi:10.3390/vetsci12080715)
Supplement: Supplementary file 1 [file vetsci-12-00715-s001.zip › FIgure S1.pdf]

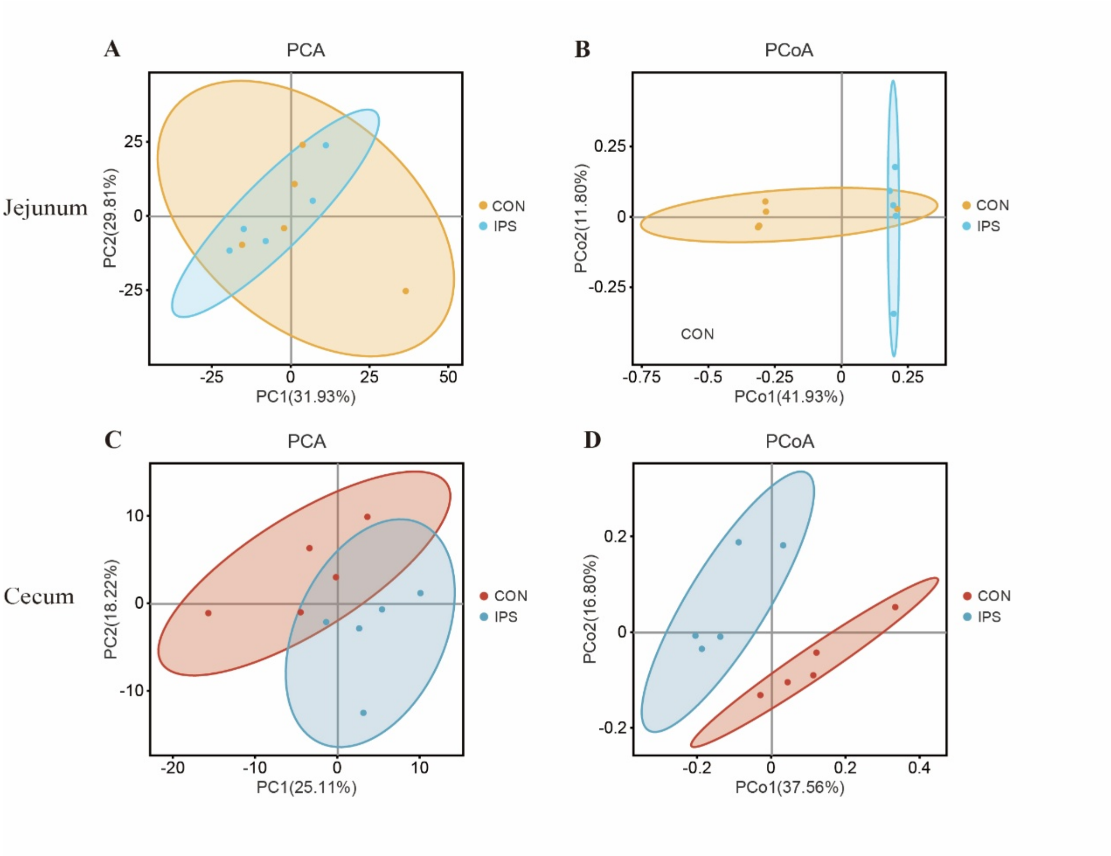

Figure S1. Microbial diversity analysis. (A) PCA of jejunal microbiota β-diversity; (B) PCoA of jejunal microbiota β-diversity; (C) PCA of cecum microbiota β-diversity; (D) PCoA of cecum microbiota β-diversity; Microbiota clustering (PERMANOVA): Cecum (R^2=0.173, *P*=0.032); Jejunum (R^2=0.092, *P*=0.671).
