# Supplementary material for: Isotonic Protein Solution Supplementation Enhances Growth Performance, Intestinal Immunity, and Beneficial Microbiota in Suckling Piglets
Source: Vet Sci. 2025 Jul 30;12(8):715. doi: 10.3390/vetsci12080715 (PMC12390416; doi:10.3390/vetsci12080715)
Supplement: Supplementary file 1 [file vetsci-12-00715-s001.zip › Table S1.pdf]

Table S1. Sequences for primers used in quantitative real-time PCR.

Gene name	Primer sequences (5′-3′)	Accession no.
β-actin	F: TCTGGCACCACACCTTCT<br>R: TGATCTGGGTCATCTTCTCAC	XM_021086047.1
ZO-1	F: AAGGTCTGCCGAGACAACAG<br>R: TCACAGTGTGGTAAGCGCAG	XM_021098896.1
Occludin	F: AACGTATTTATGACGAGCAGCCC<br>R: CACTTTCCCGTTGGACGAGTA	XM_005672525.3
Claudin-1	F: ACGGCCCAGGCCATCTAC<br>R: TGCCGGGTCCGGTAATG	AJ318102.1
IGF-1	F: CTGAGGAGGCTGGAGATGTACT<br>R: CCTGAACTCCCTCTACTTGTGTTC	NM_001097417.1
IGF-1R	F: TTCGCCAGATCCTAGGGGAG<br>R: TCCCAGCTTTGATGGTCAGG	NM_214172.1
GLP-2	F: ACTCACAGGGCACGTTTACCA<br>R: AGGTCCCTTCAGCATGTCTCT	NM_005671883.1
LBP	F: ACCGCTCCCCAGTTGGCTTC<br>R: AGCGCGGCGGACACATTAGT	NM_001128435.1
CD-14	F: TCTCACCACCCTGGACCTAT<br>R: AACTTGCGCGGACAGAGA	NM_001097445.2
MyD88	F: CTGCGTCTGGTCCATTGCTA<br>R: TGGAGAGAGGCTGAGTGCAA	NM_001099923.1
TLR 2	F: GGCAGTGTGCTGAGGAGAGAGA<br>R: CGAGGAATCATCCTGGCATT	XM_005653576.3
TLR 4	F: CCATGGCCTTTCTCTCCTG<br>R: TCAGCTCCATGCATTGGTAA	NM_001113039.2
TLR 9	F: CAATGACATCCATAGCCGAGT<br>R: CGTTGCCGCTAAAGTCCA	NM_213958.1
EREG	F: AAGACAATCCAGGTGTGGCTCAAG<br>R: CGATTTTTGTACCATCTGCAGAAA	XM_013978775.2
OASL	F: TGGTACCTGAAGTACGTGAAAGC<br>R: TACCCACTTCCCAGGCATAG	NM_001031790.1

**ZO-1*, zonula occludens-1; *CLDN-1*, claudin-1; *IGF-1*, insulin-like growth factor-1; *IGF-1R*, insulin-like growth factor-1 receptor; *GLP-2*, glucagon-likepeptide-2; *LBP*, lipopolysaccharide binding protein; *MyD88*, myeloid differentiation factor 88; *TLR 2/4/9*, toll--like receptor 2/4/9; *EREG*, epiregulin.
