# Supplementary material for: Isotonic Protein Solution Supplementation Enhances Growth Performance, Intestinal Immunity, and Beneficial Microbiota in Suckling Piglets
Source: Vet Sci. 2025 Jul 30;12(8):715. doi: 10.3390/vetsci12080715 (PMC12390416; doi:10.3390/vetsci12080715)
Supplement: Supplementary file 1 [file vetsci-12-00715-s001.zip › Table S2.pdf]

Table 2. Quality control statistics of 16S rRNA sequencing data from jejunum and caecum in piglets.

Sample Name	Raw Reads	Clean Reads	Raw Tags	Clean Tags	Chimera	Effective Tags	Effective Ratio (%)
**Jejunum**							
CON-1	134000	133892	132695	132387	15259	117128	87.41
CON-2	128430	128331	127092	126786	11177	115609	90.02
CON-3	124878	124775	123676	123325	16784	106541	85.32
CON-4	121987	121877	120628	120351	15599	104752	85.87
CON-5	127739	127616	126416	126097	14121	111976	87.66
IPS-1	127618	127489	126149	125700	15551	110149	86.31
IPS-2	123361	123272	122028	121647	15389	106258	86.14
IPS-3	120480	120391	119141	118734	10909	107825	89.5
IPS-4	131789	131668	130279	129837	9852	119985	91.04
IPS-5	131001	130894	129558	128760	17708	111052	84.77
**Caecum**							
CON-1	137276	137185	135785	135220	11302	123918	90.27
CON-2	136276	136170	134565	134084	17669	116415	85.43
CON-3	127666	127579	126021	125635	16639	108996	85.38
CON-4	122221	122113	120771	120364	15184	105180	86.06
CON-5	134311	134194	132764	132270	18192	114078	84.94
IPS-1	127186	127093	125512	125129	15001	110128	86.59
IPS-2	133288	133170	131648	131232	16690	114542	85.94
IPS-3	135072	134962	133507	133017	16705	116312	86.11
IPS-4	130516	130412	128811	128377	16818	111559	85.48
IPS-5	130357	130248	128925	128501	16034	112467	86.28
